# Supplementary material for: Phenotypic and Genomic Comparison of Staphylococcus aureus Highlight Virulence and Host Adaptation Favoring the Success of Epidemic Clones
Source: mSystems. 2022 Nov 21;7(6):e00831-22. doi: 10.1128/msystems.00831-22 (PMC9765012; doi:10.1128/msystems.00831-22)
Supplement: TABLE S3 [file msystems.00831-22-s0007.docx]

| IEC type | A sea-sak-chp-scn |
| --- | --- |
|  | B sak-chp-scn |
|  | C chp-scn |
|  | D sea-sak-------scn |
|  | E sak-------scn |
