# Supplementary figures and images for: Phenotypic and Genomic Comparison of Staphylococcus aureus Highlight Virulence and Host Adaptation Favoring the Success of Epidemic Clones
Source: mSystems. 2022 Nov 21;7(6):e00831-22. doi: 10.1128/msystems.00831-22 (PMC9765012; doi:10.1128/msystems.00831-22)

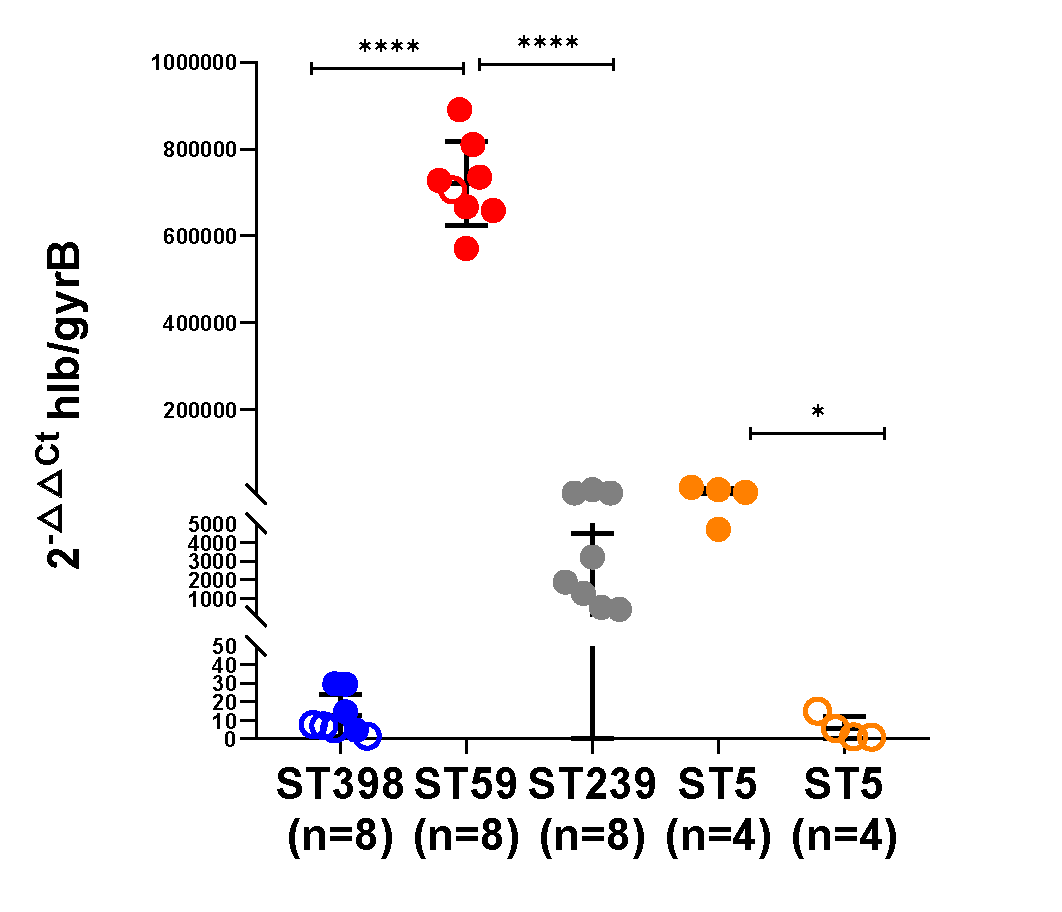

Supplement: FIG S2 [file msystems.00831-22-s0002.tif]

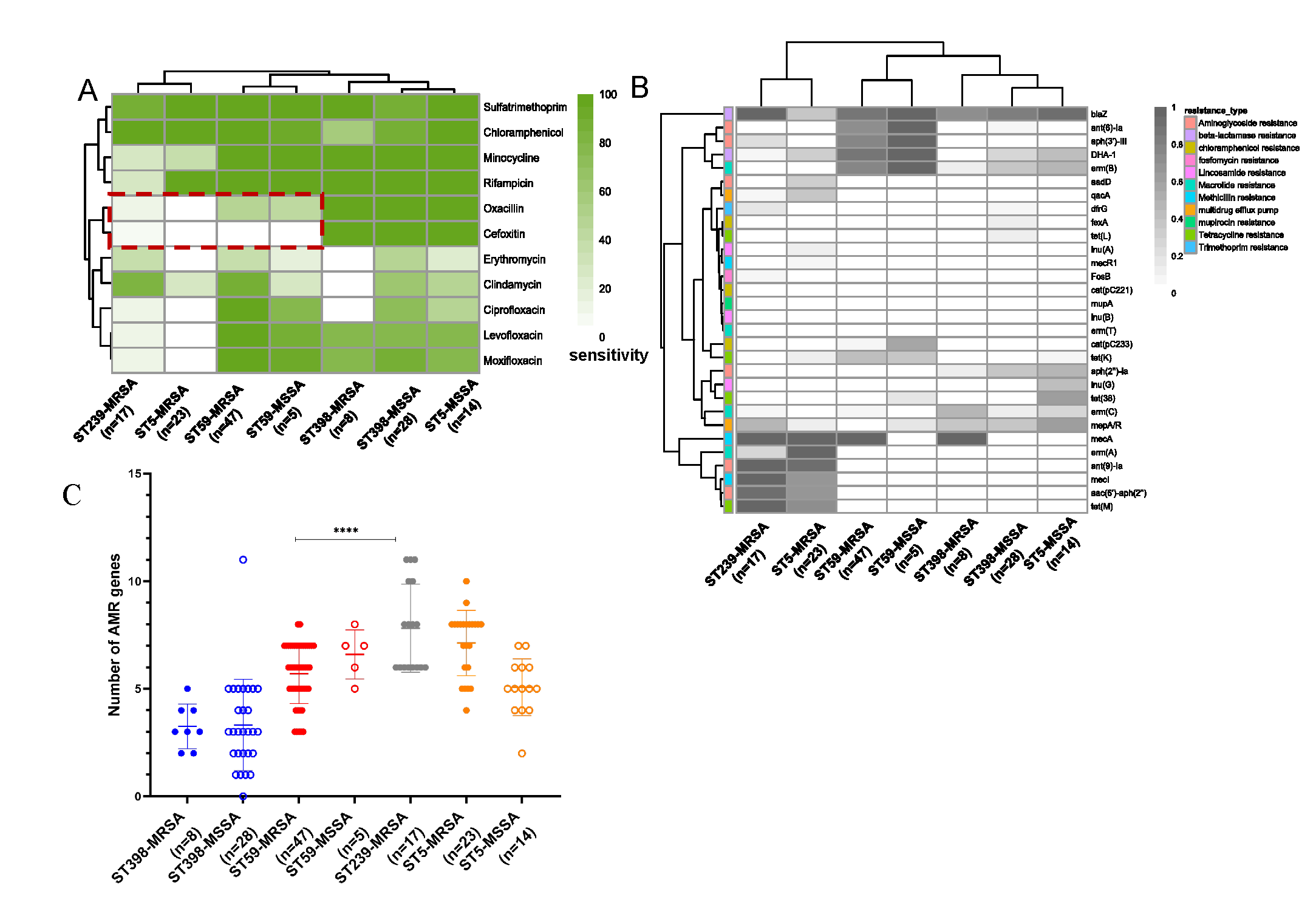

Supplement: FIG S3 [file msystems.00831-22-s0003.tif]
