# Supplementary material for: Phenotypic and Genomic Comparison of Staphylococcus aureus Highlight Virulence and Host Adaptation Favoring the Success of Epidemic Clones
Source: mSystems. 2022 Nov 21;7(6):e00831-22. doi: 10.1128/msystems.00831-22 (PMC9765012; doi:10.1128/msystems.00831-22)
Supplement: TABLE S2 [file msystems.00831-22-s0006.docx]

|  | opp-1 | opp-2 | opp-3 | opp-3' | opp-4 | opp-5 |
| --- | --- | --- | --- | --- | --- | --- |
| functions reported | cobalt and nickel transporter | nickel transporter;virulence | nitrogen utilization;peptide acquisition | unknown | unknown | nickel transporter |
| ST59 | ABCDF | BCDF | BCDFA | - | ADFBC | Opp5A |
| ST239 | ABCDF | BCDF | BCDFA | - | ADFBC | Opp5A |
| ST398 | ABCDF | BCDF | BCDFA | BCDFA | - | Opp5A |
| ST5 | ABCDF | BCDF | BCDFA | - | ADFBC | Opp5A |
