# Supplementary material for: Phenotypic and Genomic Comparison of Staphylococcus aureus Highlight Virulence and Host Adaptation Favoring the Success of Epidemic Clones
Source: mSystems. 2022 Nov 21;7(6):e00831-22. doi: 10.1128/msystems.00831-22 (PMC9765012; doi:10.1128/msystems.00831-22)
Supplement: TABLE S1 [file msystems.00831-22-s0005.docx]

| **Primer** | **Sequence (5′–3′)** | **Application** |
| --- | --- | --- |
| RNAIII_F | ATAGCACTGAGTCCAAGGAAACTAACT | qRT-PCR |
| RNAIII_R | GCCATCCCAACTTAATAACCATGT | qRT-PCR |
| psmα_F | TATCAAAAGCTTAATCGAACAATTC | qRT-PCR |
| psmα_R | CCCCTTCAAATAAGATGTTCATATC | qRT-PCR |
| hlb_F | AGCCGAATCTAAGAAAGATGATACT | qRT-PCR |
| hlb_R | AGCACGTTTATATTGCCCCCA | qRT-PCR |
| gyrB_F | CAAATGATCACAGCATTTGGTACAG | qRT-PCR |
| gyrB_R | CGGCATCAGTCATAATGACGAT | qRT-PCR |
